# Carbon-Based Nanomaterials for Plasmonic Sensors: A Review

**DOI:** 10.3390/s19163536

**Published:** 2019-08-13

**Authors:** Banshi D. Gupta, Anisha Pathak, Vivek Semwal

**Affiliations:** Department of Physics, Indian Institute of Technology Delhi, New Delhi 110016, India

**Keywords:** surface plasmon resonance, sensors, carbon nanotube, graphene

## Abstract

The surface plasmon resonance (SPR) technique is a remarkable tool, with applications in almost every area of science and technology. Sensing is the foremost and majorly explored application of SPR technique. The last few decades have seen a surge in SPR sensor research related to sensitivity enhancement and innovative target materials for specificity. Nanotechnological advances have augmented the SPR sensor research tremendously by employing nanomaterials in the design of SPR-based sensors, owing to their manifold properties. Carbon-based nanomaterials, like graphene and its derivatives (graphene oxide (GO)), (reduced graphene oxide (rGO)), carbon nanotubes (CNTs), and their nanocomposites, have revolutionized the field of sensing due to their extraordinary properties, such as large surface area, easy synthesis, tunable optical properties, and strong compatible adsorption of biomolecules. In SPR based sensors carbon-based nanomaterials have been used to act as a plasmonic layer, as the sensitivity enhancement material, and to provide the large surface area and compatibility for immobilizing various biomolecules, such as enzymes, DNA, antibodies, and antigens, in the design of the sensing layer. In this review, we report the role of carbon-based nanomaterials in SPR-based sensors, their current developments, and challenges.

## 1. Introduction

Sensors find applications in the monitoring of the environment, health, and food quality, in addition to testing and optimization of the best performance of any technique. Various spectroscopic, piezoelectric, optical, electrical, and chemical techniques have been reported in the literature for the fabrication of sensors. For example, a modified broadband dielectric spectroscopic tool, along with interdigitated electrodes, have been reported for the sensing of organic aerosols for environmental applications [1]. Similarly, a biological sensor has been reported for the sensing of dopamine (a neurotransmitter) using a photo-electrochemical method utilizing a TiO_2_ nanotube array deposited with a CdSe semiconductor [2]. In recent years, a tremendous amount of work has been reported on plasmonic sensors for the measurements of various physical, chemical, and biological parameters. Plasmonic sensors are the most powerful tools to probe and quantify low molecular analytes at low concentration levels due to their label free detection methodology, ease of operation, and high sensitivity. They have detection applications in various areas, ranging from security, environmental monitoring, food safety, pharmaceutics, and biosensing [3,4,5,6,7]. The growing research field of these sensors is now concentrated on the improvement of sensitivity and detection limit, pertaining, especially, to low concentration analytes in biological systems. The use of nanomaterials has brought considerable improvement in the order of sensitivity of plasmonic sensors [8,9]. To realize these plasmonic sensors, different types of materials have been used for their fabrication. The choice of material decides the performance of the sensor. In recent years, carbon-based nanomaterials have attracted the attention of the scientific community for the fabrication of plasmonic-based sensors. This is because of their unique electrical, mechanical, chemical, thermal, and optical properties. The present review is focused on carbon-based nanomaterials (CBNs) for their applications in plasmonic sensors.

### 1.1. Plasmonics

**Figure 1:**
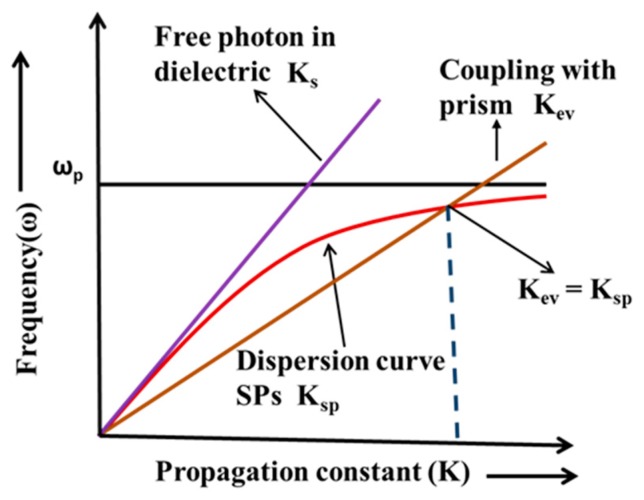
Dispersion curve for surface plasmons and incident light.

It is known that the phenomenon of oscillations plays a crucial role in the universe and is found in almost every object. The oscillations of electrons are responsible for all the interesting phenomenon in electromagnetic theory. In the 19th century, Maxwell theoretically predicted the existence of electromagnetic (EM) waves, which was later experimentally demonstrated by Hertz. In 1902, Woods reported the uneven distribution of light in a diffraction grating spectrum, known as Wood’s anomalies [10]. In 1928, Langmuir studied oscillations in ionized gas [11]. Later, Pines and Bohm stated that the energy losses experienced by fast electrons in passing through foils are due to the excitation of plasmons with the resonance frequency in bulk plasma, as [12,13,14,15]:

$$\omega_{0}=\sqrt{\frac{ne^{2}}{m\varepsilon_{0}}}$$

where *n*, *e*, and *m* are the density, charge, and mass of the electron, respectively, while *ε*_0_ is the permittivity of the vacuum. In 1957, Ritchie found that anomalous energy loss occurs in a metal thin film [16]. Later, Stern and Ferrell studied plasma oscillations and for the first time, in 1960, introduced the name surface plasmons [17]. Thus, surface plasmons are the coherent oscillations of charges at the surface of plasmonic materials. After a lot of research, it was found that the plasmons can be excited by various quantized energies, such as photons, phonons, and electrons. The coupling between the surface plasmons and photons is named surface plasmon polaritons. The solution of Maxwell’s equations for a metal/dielectric interface under appropriate boundary conditions gives the following dispersion relationship of surface plasmons [18,19,20]:

$$k_{sp}=k_{0}\sqrt{\frac{\varepsilon_{m}\varepsilon_{d}}{\varepsilon_{m}+\varepsilon_{d}}}$$

where *ε_m_* and *ε_d_* are the dielectric constants of metal and dielectric and *k*_0_ is the wave vector of light in free space. Figure 1 shows the dispersion plots of surface plasmons and direct light. It may be noted from these plots that the surface plasmons dispersion curve never intersects the dispersion curve of direct light. This implies that no phase matching or momentum matching is possible between these two wave vectors and, therefore, the surface plasmons cannot be excited by direct light. For the excitation of surface plasmons an extra momentum is required. This can be achieved by using some special structures, like prism or grating.

**Figure 2:**
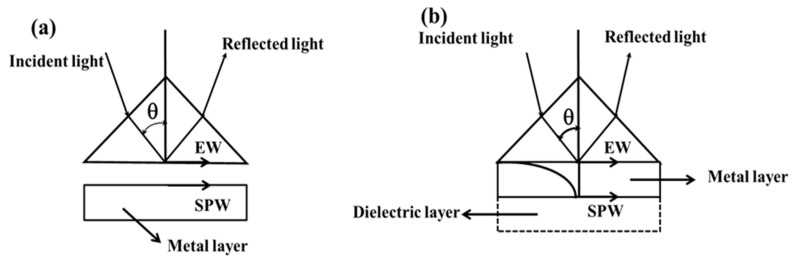
(**a**) Otto configuration, and (**b**) Kretschmann-Reather configuration.

In 1968, Otto gave a prism-based configuration where the excitation of the surface plasmons was achieved by means of an evanescent wave. In this configuration, a high index prism is kept near a metal surface, as shown in Figure 2a. The air gap between the prism base and metal surface is maintained in nanometer range. When the light incident on the base of the prism is at an angle higher than the critical angle, the total internal reflection takes place and an evanescent wave is generated at the interface of prism and air. The evanescent wave excites the surface plasmons at the interface of air and metal. In Figure 1, it can be seen that the dispersion curve of the evanescent wave intersects the dispersion curve of the surface plasmons and, at the point of intersection, the wave vector of the evanescent wave matches with the wave vector of the surface plasmon wave, resulting in the excitation of the surface plasmons. This is called surface plasmon resonance. The resonance occurs at a particular angle, called the resonance angle. At the resonance angle the reflected light intensity shows a minimum value. The maintenance of the finite gap between the base of the prism and metal layer requires a sophisticated and bulky setup. To avoid this, Kretschman and Reather modified the Otto configuration. In this configuration, a thin metal film is coated over the base of the prism and placed in contact with the dielectric medium, as shown in Figure 2b [21].

The resonance condition of surface plasmons is highly susceptible to change in the dielectric constant of the medium adjacent to the metal layer. This fact has been extensively utilised for the design of highly sensitive plasmonic sensors. They are broadly classified as propagating surface plasmon resonance (SPR) sensors or localized SPR sensors, depending on the utilization of thin film or nanomaterials of metals [18,19].

#### Kretschmann–Reather Configuration in Optical Fiber

The prism-based Kretschmann configuration requires some sophisticated instrumentation and is also bulky in size. For the compact design, the prism is replaced by the core of an optical fiber. The light inside the optical fiber is guided through the total internal reflection and the field of the evanescent wave generated decays exponentially in the cladding region. The principle for the excitation of surface plasmons is the same as in the prism-based configuration. In the case of optical fiber, cladding is removed from a small part of the fiber and the unclad core is coated with a thin metal layer. The incident light from the polychromatic source is launched from one end of the fiber and the transmitted spectrum is recorded at the other end of the fiber. A sharp dip in the spectrum is obtained at a particular wavelength, called the resonance wavelength, depending on the refractive index of the dielectric medium in contact of the metal layer [18].

### 1.2. Carbon Nanomaterials for Plasmonic Sensors

The advancement in nanotechnology during the last decade has renewed interest in every area of science and technology. The nanotechnology-enabled restructuring of existing materials at the nano-dimension has been exploited for the development of new novel materials. As mentioned in the beginning, amongst various types of nanomaterials, carbon-based nanomaterials (CBNs) have attracted significant attention from the scientific community due to their unique electrical, mechanical, chemical, thermal, and optical properties [22,23,24,25]. Carbon, with the atomic number 6, can be hybridized in sp, sp^2^, and sp^3^ states, providing various allotropes such as graphite, amorphous carbon, and diamonds, along with the newly developed superior materials like graphene, carbon nanotubes, fullerenes, and quantum dots. Graphite is the oldest and most widely available form of carbon materials, which can be given various forms with the continuously developing field of nanoscience and technology. The structural confirmation and hybridization state of carbon determine its various chemical, physical, and electronic properties in different carbonaceous materials. The basic structure in the newly developed forms of carbon is the sp^2^ bonded carbon atoms layer, i.e., each carbon atom is bonded to three other carbon atoms in same plane and a weakly delocalized π e- cloud in the perpendicular plane. This configuration is responsible for the exceptional electrical conductivity, enhanced charge transfer capability, and π-plasmon resonances in these materials. The exceptional properties of these materials have embraced the technological developments in various areas, like high tensile strength materials, catalysis, electronics and communications, biomedical applications, and sensing. The biomedical field extensively utilizes CBNs for drug delivery, therapeutics and biosensing due to their inherently favorable properties [26]. 

Amongst various carbonaceous materials, graphene and carbon nanotubes are the most widely utilized CBNs for general sensing applications. Their unique optical properties, high conductivity, which facilitates fast charge transfer reactions, high surface to volume ratio, enabling an enhancement in sensitivity, the ease of functionalization with various biomolecules and polymers for ensuring selectivity in different scenarios, chemical stability, and biocompatibility make them ideal candidates for sensing applications. Various health or biomedical, environmental, and food testing systems are reported to have utilized CBNs over the years [27,28]. There are numerous reports where graphene has been used as/for biosensing material, environmental analysis, and food quality assurance [29,30,31,32,33]. Similarly, carbon nanotubes have been extensively reviewed as an excellent material for biomedical diagnosis, the detection of many harmful environmental gases, and checking of food adulteration [34,35,36,37]. Many other carbon allotropes, such as carbon nanoparticles, carbon nanofibers, and quantum dots, are also utilized, based on various operational parameters as per the sensing application [28,29,38]. Substantial literature is available on the operational characteristics of other carbon allotropes, and therefore is not included in the present review. The present article will be focusing on carbon nanotubes and graphene-based plasmonic sensors.

CBNs, such as graphene and carbon nanotubes, are extensively used, particularly for plasmonic sensors. Thus, this review focuses on the exploitation of properties of carbon-based nanomaterials in plasmonic-based sensors [39,40,41]. Conventional noble metals, such as gold (Au), silver (Ag), copper (Cu), chromium (Cr), and aluminium (Al), are extensively used, and have for a long time been regarded as the best plasmonic materials in the fields of nanophotonics, metamaterials, and sensing. However, these materials have some shortcomings, such as large energy losses (e.g., Ohmic loss and radiative loss) and limited tunability [42,43]. Due to these shortcomings, there is a continuous surge of new materials for plasmonic applications, which have low loss, good tunability, and provide improvement in sensitivity for sensing. CBNs have emerged as the new choice of plasmonic materials, with the fulfilment of all the above properties along, with remarkable sensing properties in all types of sensor applications. In this review, the focus is mainly on graphene (and its derivatives) and carbon nanotubes for their applications in plasmonic sensors [44,45,46].

Graphene is a one carbon atom thick layer with a hexagonal honeycomb structure. Recently, graphene has emerged as an alternate plasmonic material in the terahertz (THz) to the mid-infrared range. The area of graphene plasmonics is very interesting, but there is a large mismatch between the graphene plasmons wavevector and free space light wavevector, therefore, the detection of graphene plasmons remains a challenge [47,48]. The first study of graphene plasmon resonance was based on electron spectroscopies. The electron in graphene behaves like a massless Dirac fermions, due to which it shows extraordinary properties, such as ultra-high-mobility carriers, gate-tunable carrier densities, fine structure constant defined optical transmission, long mean free path, and quantum Hall effect [49]. The confinement of the surface plasmons in the graphene is much stronger than in conventional noble metals [50]. Therefore, graphene plasmons have been used in biosensor, terahertz detector, terahertz emitter, plasmonic metamaterial, and terahertz optoelectronics. The advantages of graphene plasmons over conventional noble metals plasmons are that they have relatively low loss, high confinement, and tunability. All these advantages make graphene plasmons promising materials for the future [51,52,53,54]. 

Another interesting carbon nanomaterial is the 1D form of rolled graphene sheets, i.e., carbon nanotubes. Carbon nanotubes (CNTs) were discovered by Iijima in 1991, and since then they have emerged as the most extensively utilized nanomaterials in various research areas due to their unique properties. CNTs are graphene sheets rolled into a cylindrical structure, which are classified as single walled carbon nanotubes (SWCNTs) and multi-walled carbon nanotubes (MWCNTs), depending on the number of rolled graphene sheets. Their properties are highly influenced by their atomic structure (chirality), size, and morphological arrangement. They have remarkable mechanical strength, extremely high electrical and thermal conductivity, exceptional physical and chemical properties, ultrafast charge transfer properties, and distinct optical response in the whole EM spectrum [55]. The intrinsic relationship between their electrical, mechanical, and optical properties make them ideal for future nanoscale sensors [56]. The ultrafast charge transfer reactions and the possibility of their surface functionalization in numerous ways have made CNTs an ideal candidate for the designing of specific sensors for a plethora of applications, along with the plasmonic properties endowed by the graphitic structure [40,45,57,58]. There are numerous reports on the study of optical and plasmonic properties of carbon nanotubes, but their direct use as plasmonic material for sensing is limited due to the excitation regime in the UV or IR region. 

In plasmonic sensors, either the surface plasmon phenomenon itself is employed for sensing, or it contributes towards the enhancement of other spectroscopic methods, like surface enhanced Raman spectroscopy (SERS) and surface plasmon enhanced fluorescence [52,59,60,61,62,63,64]. Carbon nanomaterials have long been known for the enhancement of surface plasmons in several of these phenomena, however, in this review the attention is on SPR-based sensors. The role played by CBNs is basically divided in three categories for SPR sensors—plasmonic materials, sensitivity enhancement material of plasmonic sensors, and as a sensing matrix for such sensors. They are used as plasmonic materials because they are a superior substitute for noble metals, which suffer from some shortcomings, such as high ohmic losses, less tunability, and chemical instability in harsh environments. CBNs fulfill these limitations of noble metals, along with their high refractive index, which renders them with highly sensitive plasmonic properties. Due to their high refractive index, conductivity, and high surface to volume ratio, they are also utilised with noble metals for sensitivity enhancement tags in plasmonic sensors. Furthermore, their ability of numerous surface functionalization with various biomolecules, polymers, and other nanomaterials make them ideal candidates for sensing matrix in plasmonic sensors. 

## 2. Synthesis of Carbon Nanomaterials

### 2.1. Graphene and Its Derivatives

Although few-layer graphite was synthesized around 1958, the first graphene monolayer film was prepared by Novoselov, Geim, and co-workers in 2004 by mechanical exfoliation of the highly oriented pyrolytic graphite. In this technique, graphite crystal is repeatedly peeled with adhesive tape until the monolayer is found [51]. Now, many techniques and methods, like chemical vapor deposition (CVD), thermal reduction, and chemical, have been developed for this. The most popular method for the preparation of the graphene oxide (GO) and reduced graphene oxide (rGO) was reported in 1958 by Hummers, in which graphite was oxidized by KMnO_4_ and NaNO_3_ in the presence of concentrated H_2_SO_4_. Later, various modifications were made by the researchers of the Hummers method, and now it is known as the modified Hummers method. GO nanosheets are the oxidized form of graphene and can be reduced by various methods [65,66]. 

In the CVD method, the substrate is exposed to one or more precursors, with certain conditions, such as temperature and pressure, and the precursor reacts with the substrate and deposits the desired film over the substrate. Various transition-metal substrates, like Ni, Cu, Pd, and Ru, have been used for the preparation of graphene by the CVD process [67,68,69,70]. In most of the CVD growth of graphene, Ni and Cu substrates have been used. Methane gas at 900 °C exposed over Ni forms thin graphite [71]. In 2006, the first experiment to prepare graphene via the CVD method was performed, in which the precursor camphor was used on Ni foil [72]. Nowadays, various hydrocarbons, such as methane, benzene, ethylene, and acetylene, are decomposed on various transition metals substrates, such as Ni, Cu, Au, Co, and Ru, for the preparation of graphene [69].

### 2.2. Carbon Nanotubes

Three main commercially recognized production techniques for CNTs are arc discharge, laser ablation, and chemical vapor deposition (CVD) [55,73]. The arc discharge method takes place at low pressure (30–150 torr), where two high purity graphite electrodes (diameter 6–12 mm) separated by 1–2 mm are utilized as carbon sources in a chamber generally filled with helium (hydrogen and methane atmospheres are also used). The anode is sometimes mixed with metals such as Fe, Y, Mo, Co, and Ni to produce variable nature CNTs, such as graphitic, SWCNTs, metal filled CNTs, and metal nanoparticles decorated CNTs. A direct current is passed through the chamber to produce arching, where the temperature reaches up to 4000 K. Under these conditions, carbon sublimes from the anode solidify on the cathode, where a soot is formed at a typical rate of 1 mm/min. The cathode soot, chamber soot, and inner core generally consist of single and multi-walled carbon nanotubes, depending on the use of a catalyst in the anode or pure anode. Various parameters, such as reaction environment, gas pressure inside the chamber, and the type of arc discharge, control the properties of synthesized tubes. The arc discharge method is good for the large-scale production of CNTs, however it provides a very little control over its chirality [55]. The second method employed for the synthesis of CNTs is laser ablation, which can be divided into classical laser ablation and ultrafast laser ablation. Here, a metal-graphite composite rod is placed in a high temperature furnace and scanned through a high-power laser. The soot produced due to target vaporization by the laser is swept by Ar gas in the chamber and is deposited on a cooled copper target outside the furnace. The properties of the produced tubes are governed by target compositional ratio, furnace temperature, the nature of ambient gas in the furnace, and laser parameters. Laser ablation produces high purity and better graphitized CNTs than the arc discharge method, however, the mass production is limited in this technique [55]. The most important and standard method for the large-scale production of high purity carbon nanotubes is the chemical vapor deposition (CVD) method. Various modifications have been made in the standard CVD technique to produce CNTs with desired properties, such as catalytic CVD (CCVD), plasma enhanced CVD, microwave plasma assisted CVD, water assisted CVD, and radio frequency CVD. CCVD is the most extensively utilized method for the production of CNTs. Here, a catalyst is placed in a quartz boat, typically, in a horizontal flow furnace at atmospheric pressure. The reaction mixture, consisting of a hydrocarbon and an inert gas, is passed over the bed of the catalyst at temperatures ranging from 500–1100 °C. The CNT growth in such a procedure involves the dissociation of hydrocarbon in the presence of transition metal catalysts and the saturation of carbon atoms in metal particles in an sp^2^ structure in a tubular carbon state. The properties depend on various parameters, like temperature, pressure, type and concentration of hydrocarbons and metal catalysts, and the reaction time [73]. Some other less popular methods also employed for the production of CNTs involve the hydrothermal method and electrolysis. 

## 3. Carbon Nanomaterials-Based Plasmonic Sensors

In plasmonic sensors, carbon nanomaterials have been used (i) to act as a plasmonic material, (ii) as the sensitivity enhancement material, and (iii) as a sensing matrix. We shall discuss, below, all the three major roles one by one. 

### 3.1. Carbon Nanomaterial as Plasmonic Material

#### 3.1.1. Graphene

Recently, a graphene-based plasmonic refractive index sensor has been theoretically investigated. The sensor works in the mid-infrared region at room temperature and graphene plasmon frequency shifts for a small change in the refractive index around the graphene sheet have been observed. The resonance wavelength was found to increase with an increasing refractive index of the medium around the graphene [74]. The sensor possesses a sensitivity of 2.5 µm/RIU, which means the resonance wavelength shifts by 2.5 µm for every unit change in refractive index. Here, RIU means refractive index unit. The figure of merit (FOM) of the sensor was reported to be around 10.7. Similar to this, various graphene-based plasmonic sensors have been reported in the literature for different applications [5,74].

#### 3.1.2. Carbon Nanotubes

Carbon nanotubes have also been explored as plasmonic materials for sensing in numerous applications. In a localized surface plasmon resonance (LSPR)based gas sensor, CNTs are utilized as plasmons, as well as an affinity material for the detection of carbon dioxide (CO_2_) gas [75]. CNTs are believed to show a change in their electrical properties, which alters their optical properties upon chemical interaction with CO_2_. This property is exploited to fabricate an optical fiber Bragg grating-based LSPR sensor. The authors successfully showed a sensitive response with the observation of a direct change in optical response of CNTs upon chemical interaction with a molecule. A refractive index sensitivity (Δλ/Δn) of 6200 nm/RIU in the gaseous regime was observed, which contributes to a detection limit of 150 ppm at atmospheric pressure, making the approach highly suitable for practical CO_2_ sensor application. The response of the probe was tested for other similar hydrocarbon gases, such as methane and ethane, for the specificity of the approach and the results were found to be highly satisfactory, with a change in SPR response only in the case of CO_2_ [75]. 

In another approach, the π plasmon absorption of carbon-based nanomaterials in the UV-region was utilized for SWCNTs for a sensitive and selective binding of ions [76]. It was observed that the strong π plasmon absorption of SWCNTs in the UV region is far more sensitive to plasmonic properties in visible and NIR regimes. The method, based on UV-Vis absorption, was found to be simple and effective as compared to previously reported field effect transistor (FETs) and fluorescence-based methods, which require complex device fabrication, NIR source, and the separation of semiconducting and metallic CNTs. It was also found to be highly specific for the detection of metal ions by tailoring the surface modification of CNTs through molecular legends specific to ion attachment. The model was tested to detect Fe^3+^ ions in an approach based on the surface modification of SWCNTs by a natural bacterial siderophore, Deferoxamine (DFO), for the specific attachment. 

**Figure 3:**
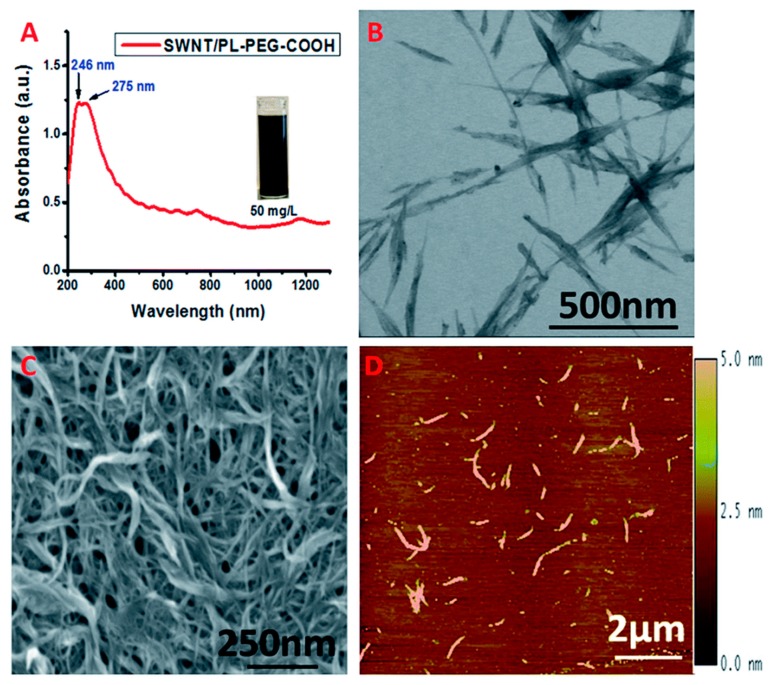
(**A**) UV-Vis-NIR spectrum of SWNT/PL-PEG-COOHs in aqueous solution. (**B**–**D**) STEM, SEM and AFM images of SWNT/PL-PEG-COOHs, respectively. Reprinted with permission from [76]. Copyright 2016 Royal Society of Chemistry.

**Figure 4:**
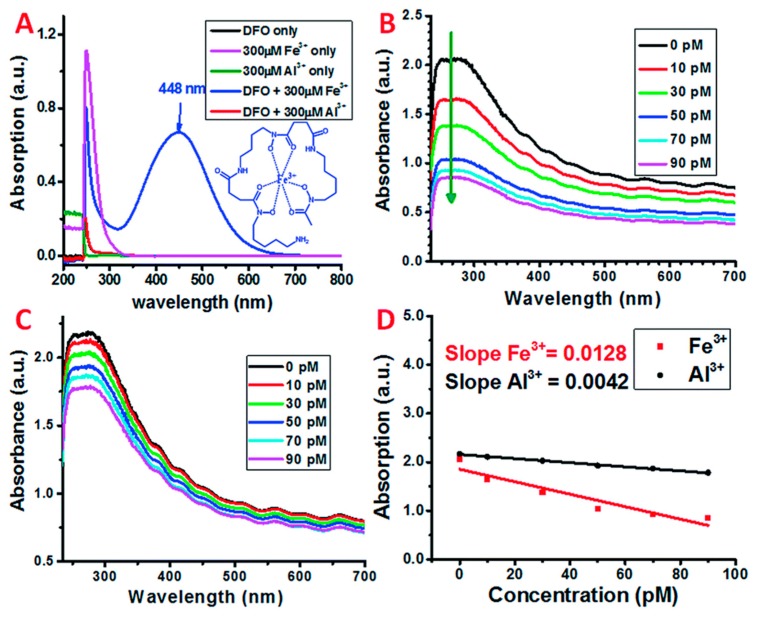
(**A**) UV-Visible spectra of Deferoxamine (DFO) only, Fe^3+^ only, Al^3+^ only, DFO + Fe^3+^, and DFO + Al^3+^ solution in nitric acid (pH = 2). (**B**,**C**) UV-Vis spectra of SWNT/PL-PEG-DFO in the presence of (black) 0 pM, (red) 10 pM, (green) 30 pM, (blue) 50 pM, (cyan) 70 pM, and (magenta) 90 pM Fe^3+^ and Al^3+^ standard solutions, respectively. (**D**) The decrease in the absorption of SWNT/PL-PEG-DFOs at 270 nm as a function of the increase in the concentration of Fe^3+^ and Al^3+^. UV-Vis-NIR spectrum. Reprinted with permission from [76]. Copyright 2016 Royal Society of Chemistry.

The CNT surfaces were first modified with carboxyl terminated phospholipid-polyethylene glycol (PL-PEG-COOHs) for stable aqueous dispersions and further attachment of siderophore. These PL-PEG-COOHs modified CNTs show two absorption peaks in the UV region, at 246 and 275 nm, due to the π-plasmon absorption by the graphitic structure, as shown in Figure 3. The detection limit reaches the pM range, with the sensing mechanism based on the charge transfer between Fe-DFO complex and SWNTs, as obtained from resonant Raman scattering study. The UV-Vis sensing results are shown in Figure 4. The testing was performed in aqueous and rain water samples, with a specific response towards Fe^3+^ ions, as tested in an interfering environment from other ions, such as Al^3+^, Zn^2+^, Ni^2+^, and Cu^2+^ (Figure 4D).

### 3.2. As Sensitivity Enhancement Material

Carbon-based nanomaterials, specifically graphene and carbon nanotubes, are widely known to be sensitivity enhancement materials for plasmonic sensors [77,78]. Therefore, they are extensively reported in SERS signal enhancement substrates due to plasmon coupling with metal nanoparticles [61,63]. However, we have constrained ourselves here to discussing only the SPR-based biosensors for sensitivity enhancement due to these carbonaceous materials.

#### 3.2.1. Graphene and Its derivatives

**Figure 5:**
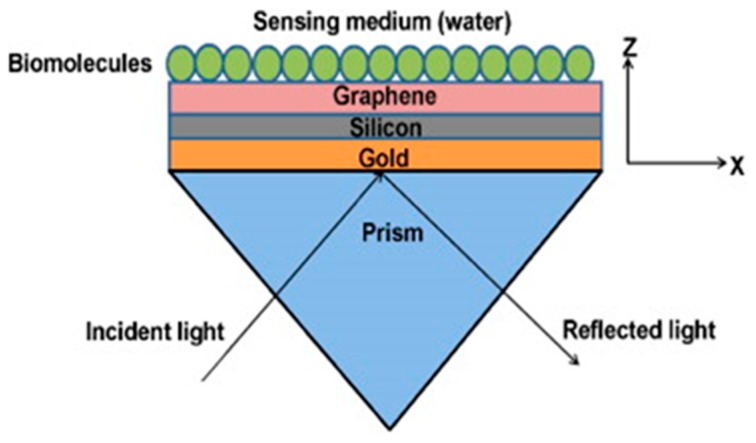
Schematic diagram of a prism based SPR probe. Reprinted with permission from [77]. Copyright 2011 Elsevier.

In recent years, a number of plasmonic sensors have been proposed using graphene and its derivatives, with plasmonic metal layers for the purpose of sensitivity enhancement. A theoretical study of surface plasmon resonance-based biomolecules sensors using a graphene layer has also been reported. In this study, the graphene layer was used over the gold and silicon layer to enhance the sensitivity of the sensor [77]. The schematic of a prism-based SPR sensor is shown in Figure 5. The sensor model consisted of coatings of gold, silicon, graphene, and biomolecule layers over the base of the prism. The gold was used as a plasmonic layer, while the silicon and graphene layers were used for sensitivity enhancement. Apart from sensitivity enhancement, the graphene layer also helps in the enhancement of the immobilization of the biomolecules. The thicknesses of gold, silicon, and graphene layers were optimized via simulation. The best sensitivity of the sensor was achieved for a 40 nm thick gold layer, 7 nm thick silicon layer, and two layers of graphene at 633 nm wavelength of the light source. 

**Figure 6:**
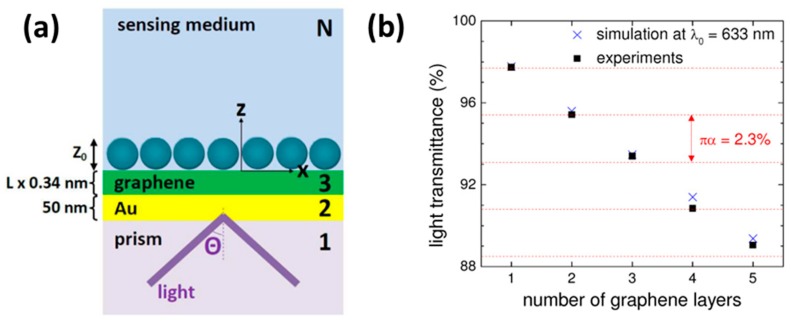
(**a**) The N-Layer model for surface plasmon resonance (SPR) biosensor: prism|Au (50 nm)|graphene (L× 0.34 nm)|sensing medium, and (**b**) simulated transmittance of light at λ_o_ = 633 nm (crosses) and measured transmittance of white light (squares) as a function of the number of graphene layers. Reprinted with permission from [79]. Copyright 2010 The Optical Society.

Another experimental and theoretical study utilizing graphene layer over the gold layer has been reported for the sensitivity enhancement of an SPR biosensor. In this study, the prism was coated with a 50 nm thick gold layer, followed by coatings of graphene and biomolecules (DNA) layers [79]. The layer configuration is shown in Figure 6a. For the simulation of a graphene-based SPR sensor, an N-layer model was used and the results were compared with the experiments. In the simulations, the complex refractive index of the graphene (*n*) was taken as:

$$n=3+i\frac{c}{3}\lambda$$

where *λ* is the wavelength of light and *c* is a constant. The thickness of a single graphene was taken as 0.34 nm.

**Figure 7:**
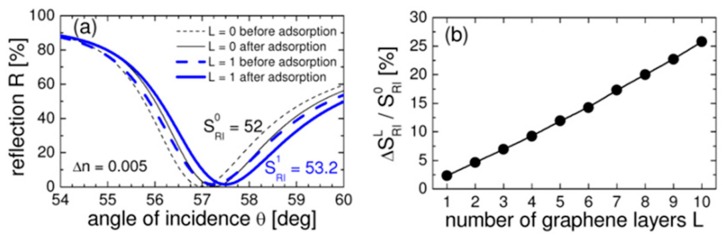
(**a**) The surface plasmon resonance curves for the conventional biosensor and the monolayer graphene biosensor for the He-Ne laser light, and (**b**) the sensitivity enhancement as a function of the number of graphene layers. Reprinted with permission from [79]. Copyright 2010 The Optical Society.

Figure 6b shows the percentage of transmitted light with the number of graphene layers. From this graph, it is clear that the light transmitted through the monolayer graphene is around 97.7% and the transmittance decreases with the increasing graphene thickness. The experimental data shows good agreement with simulated data. Figure 7a shows the surface plasmon resonance curve for the conventional biosensor and monolayer graphene biosensor. From the figure, it is clear that the SPR angle shifts more in the case of the monolayer graphene biosensor. Figure 7b shows the sensitivity enhancement with the number of graphene layers.

The improvement in sensitivity was due to two reasons. First, the graphene strongly absorbs the biomolecules with a carbon-based ring structure, which enhances the absorption efficiency of the biomolecules, and second, the optical properties of the graphene modify the SPR curves and provide a large shift for the refractive index change. In summary, this study shows that a graphene-based biosensor enhances the sensitivity 2.5 times in comparison to a conventional biosensor.

**Figure 8:**
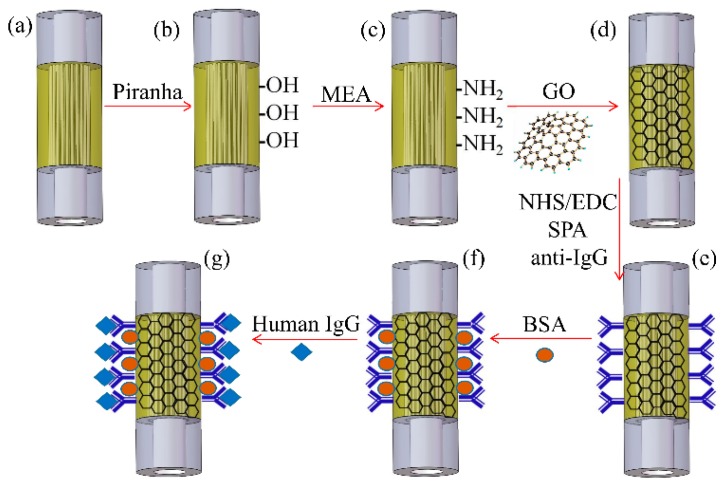
Procedure of graphene oxide and staphylococcal protein A (SPA) modification for IgG immunoassay. Reprinted with permission from [80]. Copyright 2018 Elsevier.

An experimental study on the enhancement of sensitivity of an SPR-based sensor using graphene oxide and biomolecules attachments has also been reported. In this study, the sensitivity of an SPR-based photonic crystal fiber (PCF) refractive index sensor was compared with an immunosensor with and without a graphene oxide layer. The probe was designed by splicing the PCF fiber between the two multimode fibers [80]. To fabricate the sensor, the 5 mm sensing length of the PCF fiber was first coated with a 5 nm thin chrome layer and then with a 50 nm thin film of gold. After this, an over-layer of graphene oxide was deposited over the gold layer through various steps, as shown in the Figure 8a–d. For the immunosensor, the staphylococcal protein A (SPA) was attached over the GO surface, as shown in Figure 8e–g.

**Figure 9:**
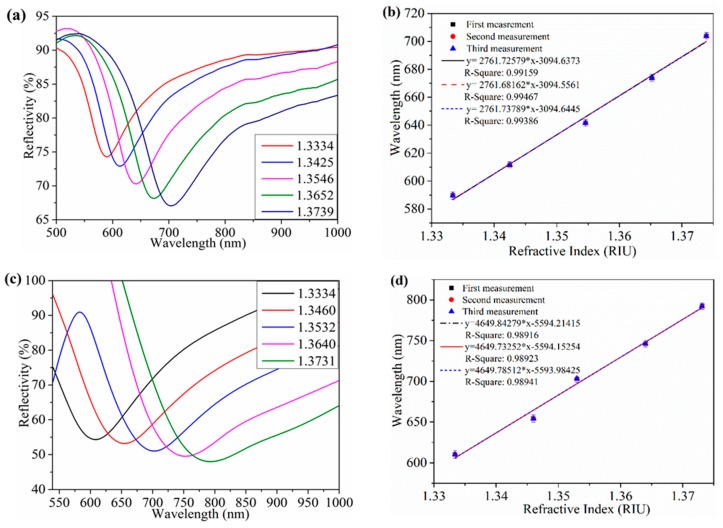
The resonance spectrum and linear fitting curve of Au-PCF (**a**,**b**) and Au/GO-PCF (**c**,**d**) sensor. The errors bars represent the standard deviations from three independent experiments. Reprinted with permission from [80]. Copyright 2018 Elsevier.

The SPR spectra for the gold coated PCF fiber (Au-PCF) and gold and graphene oxide coated PCF fiber (Au/GO-PCF) are shown in Figure 9a,c, for the refractive index range from 1.33 to 1.37. The shift in the resonance wavelength corresponding to Au-PCF and Au/GO-PCF sensors for the change in the refractive index are shown in Figure 9b,d. 

The change in the resonance wavelength for the Au-PCF and Au/GO-PCF sensors were reported to be 114 nm and 181 nm for the refractive index range from 1.33 to 1.37, respectively. For the case of the Au-PCF and Au/GO-PCF sensors, the values of sensitivity obtained were 2761.7 nm/RIU and 4649.8 nm/RIU, respectively. Here, the graphene oxide film enhances the interaction between the Au film and analyte which increases the refractive index sensitivity.

**Figure 10:**
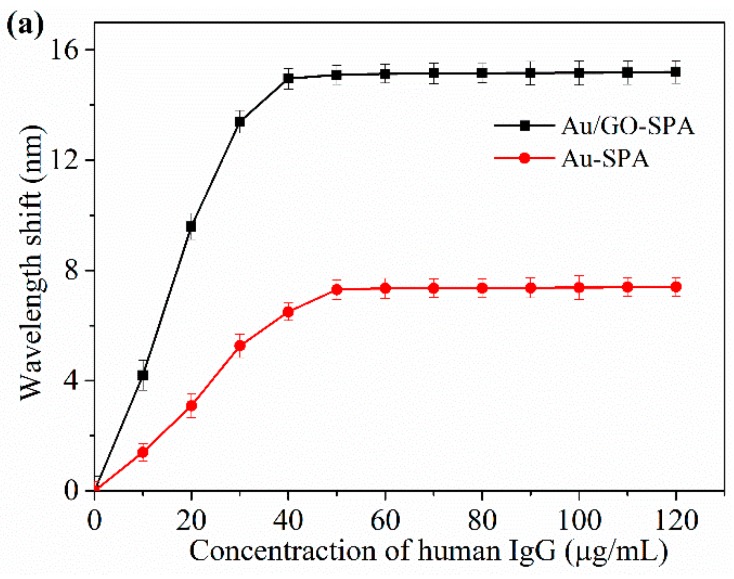
Goat anti-human IgG immobilized on the sensor surface. Reprinted with permission from [80]. Copyright 2018 Elsevier.

For the SPR immunosensor, the attachment steps of SPA are represented in Figure 10. Figure 10 shows the wavelength shift curve of anti-human IgG immobilized on the Au/GO-SPA and Au-SPA sensor. When the IgG sample comes in contact with the sensor surface, the refractive index changes and a red shift is observed. The change in the resonance wavelength for the Au-SPA sensor is around 7.4 nm, while for the Au/GO-SPA sensor it is 15.2 nm. This enhancement shows that due to the large surface area and biocompatibility, GO allows for more immobilization of the antibody than the gold surface, which leads to the larger resonance wavelength shift. This study shows that GO has a great potential in sensitivity enhancement for SPR-based refractive index sensors or biosensors.

**Figure 11:**
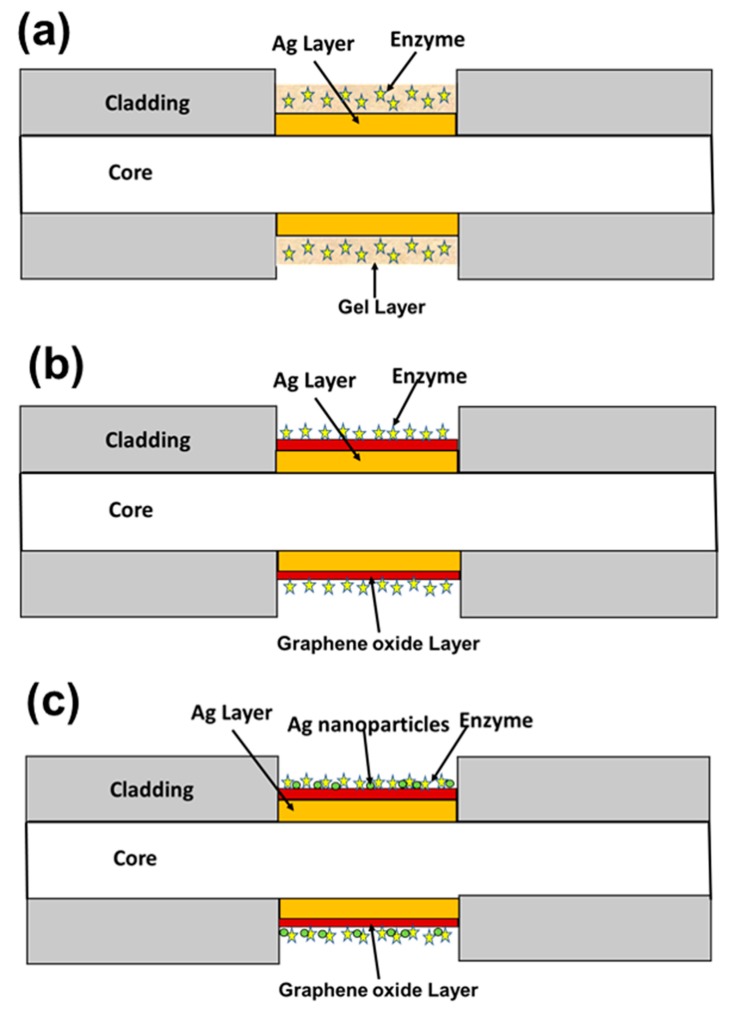
Schematic diagram of three different fiber optic probes, named (**a**) Probe I, (**b**) Probe II, and (**c**) Probe III.

In another study, an SPR-based fiber optic cholesterol sensor with three different approaches was discussed [81]. In this study, three different types of probes were fabricated, as shown in Figure 11. In probe I, the gel entrapment method was used for the detection of cholesterol, while in probe II, the enzyme cholesterol oxidase (Chox) was immobilized over the graphene oxide nanosheets, and in probe III, polyvinyl alcohol (PVA) embedded silver nanoparticles were decorated over the GO nanosheets. For the synthesis of GO, the modified Hummer’s method was used. In probe I, the probe was fabricated by coating a thin silver layer over the core of an optical fiber, followed by the enzyme entrapped hydrogel. Probe II consisted of a GO layer over the silver coated unclad core of the fiber, and a Chox enzyme was immobilized over it. Finally, probe III consisted of layers of silver and GO over the core of an optical fiber, followed by layers of PVA embedded silver nanoparticles and enzyme Chox. The SPR curves for probe I for a cholesterol concentration range of 0 to 10 mM shifted by around 18 nm, which gave 1.789 nm/mM as the sensitivity of the sensor. The reason behind the shift in resonance wavelength is that when the cholesterol sample interacts with the cholesterol oxidase, the enzymatic reaction takes place and it gets converted into Cholest-4en-3-one and hydrogen peroxide. Due to this enzymatic reaction, the effective refractive index of the sensing layer changes, which results in the shift in resonance wavelength. For probe II, the total shift in the resonance wavelength was around 32 nm, which was greater than that in the case of probe I. In probe II, GO provided a large surface area, and therefore the available surface area for the enzyme was increased and the shift in resonance wavelength was increased. 

Apart from their large surface area, GO nanosheets have various oxygen functional groups, such as -OH, -COOH, which help to bind the enzyme. The total shift in resonance wavelength and sensitivity for probe III were around 50 nm and 5.06 nm/mM, respectively, which were the maximums among all three probes. In probe III, the shift was due to an enzymatic reaction, as well as the silver nanoparticles, which decompose the hydrogen peroxide, due to which the effective refractive index changes more in comparison to the probe I and II, which results in the maximum shift and sensitivity. From all three probe configurations, it was concluded that GO enhances the sensitivity of the biosensor.

#### 3.2.2. Carbon Nanotubes

**Figure 12:**
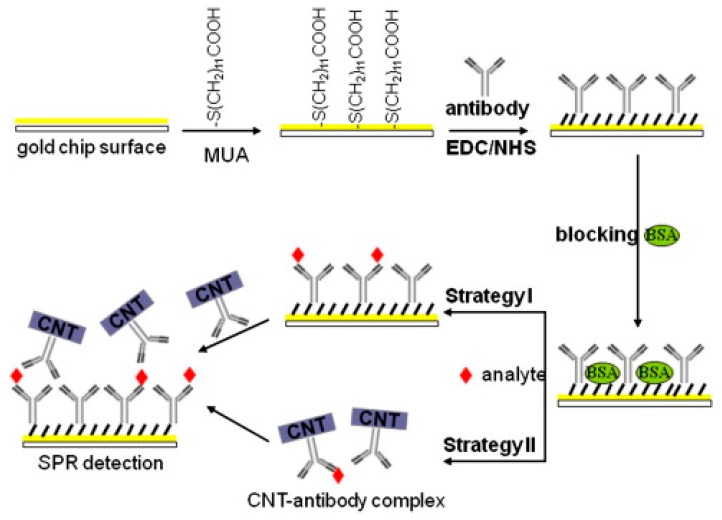
Schematic diagram of SPR immunoassay to enhance sensitivity with the CNT-antibody complex. Reprinted with permission from [82]. Copyright 2011 Elsevier.

Carbon nanotubes have also been used to enhance the sensitivity of a biosensor. In a sensitivity enhancement approach, a CNT-antibody conjugate was employed to amplify the SPR signal for a biosensing platform [82]. Human erythropoietin (EPO) and human granulocyte macrophage colony-stimulating factor (GM–CSF) were tested as model systems to see the role of CNTs in the enhancement of SPR signal. Biocompatibility, fast electron transfer, and large molecular mass were reported to be key factors for sensitivity enhancement for the detection of very low levels of EPO and GM-CSF in real systems. All SPR measurements were performed on the commercially available BIAcore X SPR biosensor system (GE Healthcare, Sweden) at a flow rate of 5 µl/min, at 25 °C. Gold chips were modified with a self-assembled monolayer of 11-mercaptoundecanoic acid (MUA) and then activated by ethyl (dimethylaminopropyl) carbodiimide /N-hydroxysuccinimide (EDC/NHS) chemistry for the capture of respective antibodies. A schematic of the detailed mechanisms of CNT assisted and without CNTs SPR detection is shown in Figure 12.

**Figure 13:**
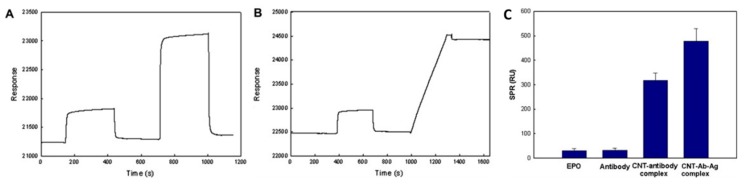
SPR sensorgrams of the (**A**) EPO (1 μg/mL) immunoassay with the antibody, (**B**) CNT–antibody complex, and (**C**) SPR signal shift by direct and CNT-mediated detections. Reprinted with permission from [82]. Copyright 2011 Elsevier.

The enhancement in SPR signal with the CNT assisted approach can be seen in Figure 13, through the real-time SPR response and the SPR angle shift in the case of direct and CNT assisted detection. The CNT-based amplification method was reported to be sensitive for a wide dynamic range, from 0.1 to 1000 ng/mL, with the detection limit reaching 0.1 ng/mL, enabling its application in real scenarios.

**Figure 14:**
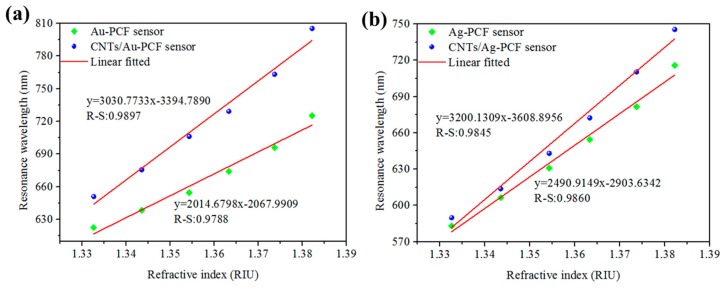
SPR resonance wavelength of (**a**) Au-PCF sensor and CNTs/Au-PCF sensor (**b**) Ag-PCF sensor and CNTs/Ag-PCF sensor with varied refractive indices of glucose solutions. Reprinted with permission from [83]. Copyright 2018 Elsevier.

In a similar approach, a photonic crystal fiber (PCF)-based SPR sensor was reported with enhanced refractive index sensitivity, due to CNTs deposited on the Au and Ag film as compared to a bare Ag/Au thin film [83]. The sensitivity of the CNT-Au film PCF SPR sensor was reported to increase by 1016 nm/RIU as compared to the conventional Au film PCF SPR sensor, whereas the sensitivity of the CNT-Ag film PCF SPR sensor increased by 709 nm/RIU compared to the Ag film SPR sensors. The experimental results were found to be in accordance with simulated data for such sensors, clarifying the role of CNTs in the sensitivity enhancement of SPR sensors. The results are shown in Figure 14.

A gold nanoparticle-decorated CNTs platform was developed recently for the LSPR based sensing of bovine growth hormone [84]. Due to the high surface to volume ratio and favorable electron transfer reactions, CNTs were augmented with Au nanoparticles to provide synergistic effects to LSPR sensing. The properties of the unique nanocomposite were explored to absorb and sense the biomolecular interaction with increased sensitivity, as compared to individual material. A concentration as low as 1 ng/mL of rbST was detected with the composite based LSPR platform, providing a new window for ultrasensitive plasmonic detection of polypeptides and proteins.

### 3.3. As sensing Matrix Material

#### 3.3.1. Graphene and Its Derivatives

**Figure 15:**
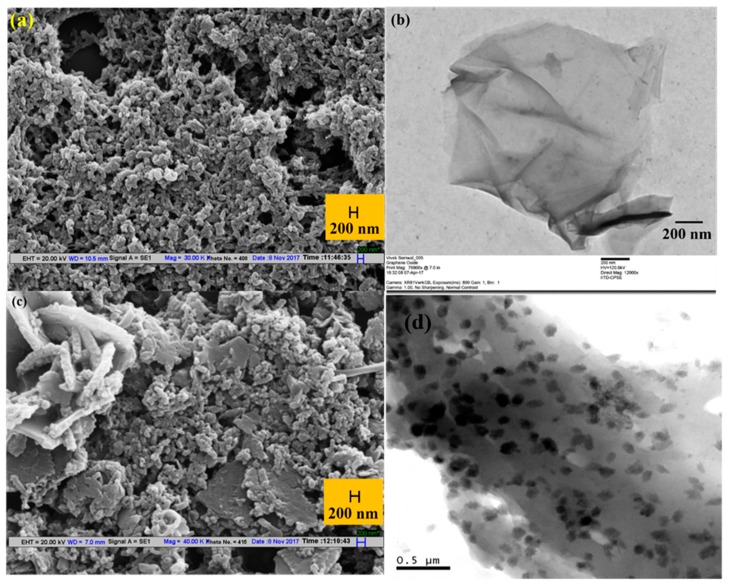
(**a**) SEM image of Pani, (**b**) TEM image of rGO, (**c**) SEM image of the rGO-Pani nanocomposite, and (**d**) HR TEM image of rGO-Pani nanocomposite. Reprinted with permission from [85]. Copyright 2019 Elsevier.

In recent years graphene, GO, reduced graphene oxide (rGO), and their nanocomposites have been extensively exploited for various biosensors, gas sensors, and chemical sensors. Graphene and its nanocomposites act as a sensing layer in various sensors, including plasmonic sensors. Since the plasmonic sensors are very sensitive to the change in the refractive index of the surrounding medium, if there is a possibility of an interaction between graphene and analyte there will be a possibility of its sensing. In such a case, one can develop a sensor using graphene and its nanocomposites. Based on this approach, a pH sensor was reported utilizing rGO and polyaniline (PANI) as sensing layers [85]. As is known, pH is a very important parameter and it plays a vital role in biological processes, drinking water, environment, food quality control, biochemistry, and chemical reactions. The study has utilized surface plasmon resonance on optical fibers. The rGO-Pani nanocomposite was prepared by an in situ method. Figure 15a shows the scanning electron microscope (SEM) image of Pani, in which the chain-like porous structure of Pani can be seen. Figure 15b represents the transmission electron microscope (TEM) image of rGO which depicts a very thin sheet of rGO. Figure 15c shows the SEM image of the rGO-Pani nanocomposite and Figure 15d shows the TEM image of the rGO-Pani nanocomposite. In this figure, the lighter portion in the image represents the rGO nanosheets and the darker part represents Pani.

**Figure 16:**
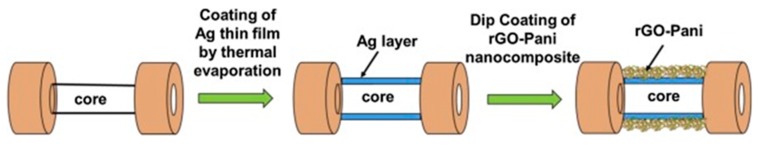
Schematic representation of the probe fabrication. Reprinted with permission from [85]. Copyright 2019 Elsevier.

The design of the sensor probe is shown in Figure 16. The unclad core of an optical fiber, in the middle, was coated with a thin silver film by the thermal evaporation method, over which another coating of rGO-Pani was done by the dip coating method.

**Figure 17:**
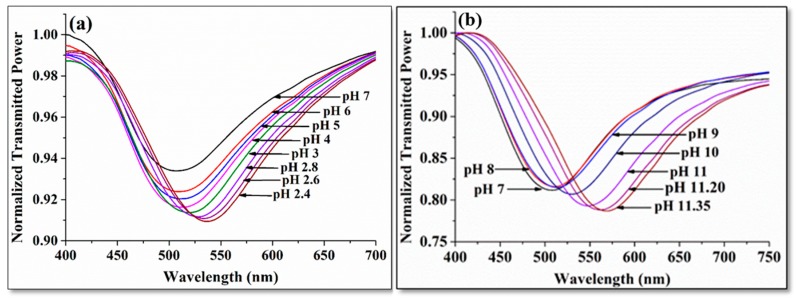
SPR spectra of the probe for (**a**) pH range from 7.0 to 2.4, and (**b**) pH range from 7.0 to 11.35. Reprinted with permission from [85]. Copyright 2019 Elsevier.

Figure 17a shows the SPR spectra for the acidic region from pH 7 to pH 2.4. As one moves towards the acidic region from pH 7, a red shift is observed in resonance wavelength. The reason behind the red shift is the chemical interaction between the rGO-Pani nanocomposite with the acidic pH samples. When an acidic sample comes into contact with polyaniline, the emeraldine form of Pani changes to pernigraniline base through an oxidation process, which changes the refractive index of Pani. Since an acidic sample has an excess of H^+^ ions, when it comes in contact with rGO nanosheets, the rGO nanosheets get converted to the n-doped material, which alters the band gap of the rGO nanosheets. Thus, both the materials in the nanocomposite are sensitive to the acidic sample and, therefore, the change in the effective refractive index of the nanocomposite is the result of change in both the materials, and hence one obtains a cumulative effect on the red shift in resonance wavelength. The sensitivity of the sensor is defined as the change in resonance wavelength per unit of change in the pH of the sample. For the acidic range, the maximum sensitivity of the sensor was found to be 24.93 nm/pH, which means the resonance wavelength shifts around 24.93 nm, when the pH of the sample is changed by unity. As reported, the exponential nature of the sensitivity plot is due to the H^+^ ions, which increase exponentially with the decrease in pH of the sample.

Figure 17b shows the SPR curves corresponding to the basic region from 7 pH to 11.35, and in this case also a red shift in the resonance wavelength was observed with the increase in pH of the sample (increase in alkanity). Again, the reason for the red shift is the interaction between the pH sample and rGO-Pani nanocompoite. The emeraldine form of Pani changes to a leucoemeraldine base after the interaction with the basic sample through the reduction process. The samples with pH from 7 to 11 have an excess of OH^−^ ions. The rGO nanosheets get converted to p-doped material via OH^−^ ions. Therefore, the overall band gap of the nanocomposite changes, which results in a change in the effective refractive index of the sensing layer. The maximum sensitivity was found to be around 75.09 nm/pH at the pH value 11.35. In this study, the behavior of rGO nanosheets changed when they came in contact with acidic/basic samples, resulting in a change in the effective refractive index. Thus, rGO nanosheets act as a sensing matrix for the measurement of the pH.

#### 3.3.2. Carbon Nanotubes

**Figure 18:**
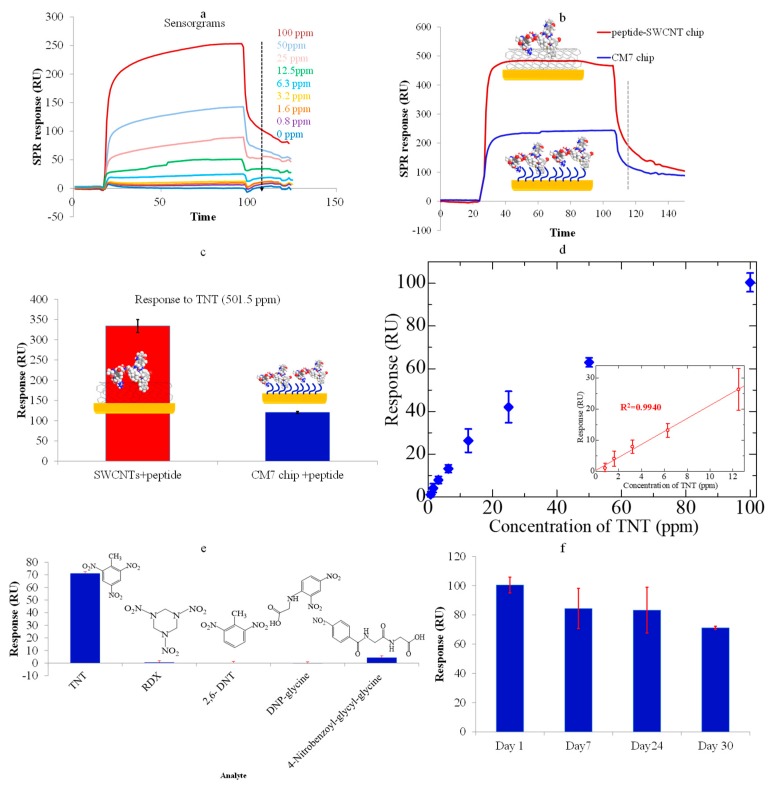
The real-time sensorgrams of (**a**) peptide-SWCNT hybrids corresponding to TNT concentrations; (**b**) two kinds of sensor chips corresponding to TNT explosives at a concentration of 501.5 ppm; (**c**) a comparison of the responses of two kinds of sensor chips; (**d**) the responses of the SWCNT–peptide chip and CM7 chip to TNT; (**e**) the response of the TNTHCDR3-anchored SWCNT sensor chip to 100 ppm solutions of TNT, RDX, 2,6-DNT, 4-nitrobenzoyl-glycyl-glycine, and DNP-glycine. (**f**) The stability of the sensor chip over a duration of one month. Reprinted with permission from [90]. Copyright 2018 MDPI.

The last way of utilizing CNTs in plasmonic sensors is as sensing material. The ease and variety of possible functionalization on CNTs side walls and edges make them suitable for the sensing of various species [86,87,88,89]. A 2,4,6-trinitrotoluene (TNT) SPR sensor was reported, with peptide modified SWCNTs as sensing material over Au-coated SPR chips. The standard gold coated chips were firstly modified with APTES (3-aminopropyltriethoxysilane) to generate amine groups for the attachment of carboxyl terminated CNTs [90]. After this, TNTHCDR3 peptide was immobilized non-covalently on CNTs through a π-π interaction. The SPR measurements were carried out on a standard Biacore system. TNT samples were tested for a wide dynamic concentration range, from 0.8 ppm to 100 ppm. The real-time SPR response of peptide modified SWCNTs based SPR sensors for various concentrations of TNTs is shown in Figure 18a. The response was compared with a conventional dextran chip (CM7) and it was revealed that CNTs offer promising properties for the detection of low molecular weight analytes at low concentration levels in SPR sensors, with a significantly improved response (Figure 18b,c). The response of the CNT modified chip is shown in Figure 18d for various concentrations of TNT. The specificity of the chip was tested for various analytes, which can interfere or are analogous to TNTs, such as DNP-glycine, 2,6-DNT, RDX, and 4-nitrobenzoyl-glycyl-glycine, showing a negligible response from all the other analytes (Figure 18e). The stability and reversibility of surface reactions on CNTs and the chemical stability of the gold chip are visible through the stable response of the sensor over 20–30 days (Figure 18f).

**Figure 19:**
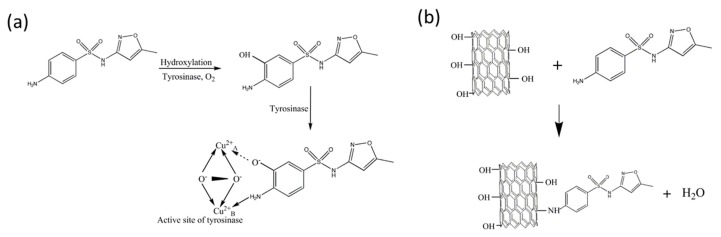
Interaction mechanism of (**a**) Tyrosinase and SMX, (**b**) functionalized CNTs and SMX. Reprinted with permission from [91]. Copyright 2018 Springer.

In another study, an optical fiber-based SPR sensor was reported, where MWCNTs were utilized as sensing material for the detection of sulfamethaxazole (SMX), opening the potential sensing applications of CNTs in pharmaceuticals [91]. The response of the sensor was compared with a similar approach based on enzyme entrapped gel as a sensing layer for a 0 to 200 µM concentration range of SMX. The CNT-based platform offers an improved sensitivity over the desired concentration range, along with long time stability and cost effectiveness as compared to the enzymatic approach. The CNT-based probes were prepared by depositing Ag thin film on the fiber core surface, functionalizing the Ag surface with APTES, and then attaching carboxylic acid functionalized CNTs on amino group-modified fiber substrate. The other enzyme-based probes were also prepared by the coating of polyacrylamide gel entrapped with tyrosinase enzyme for the sensing of SMX. The interaction mechanism governing the SPR response due to physicochemical interaction of SMX with CNTs and tyrosinase is shown in Figure 19.

**Figure 20:**
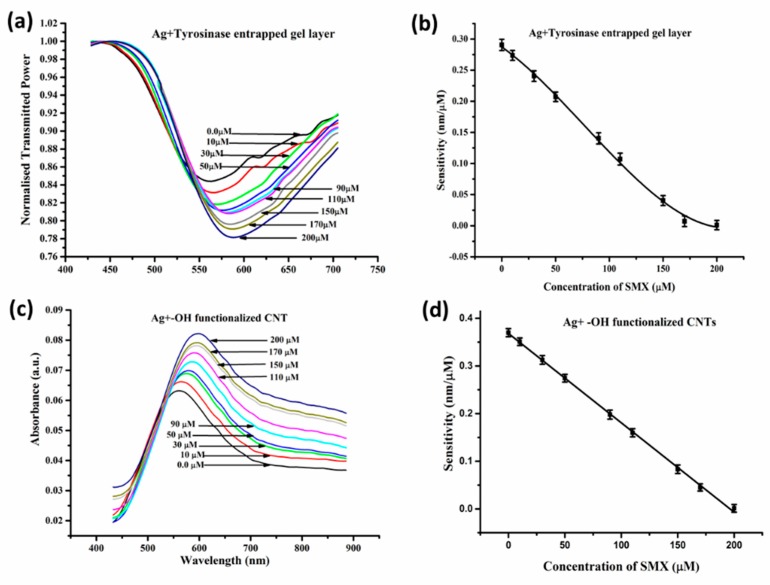
(**a**) SPR spectra, (**b**) sensitivity of the tyrosinase enzyme-based probe, and (**c**) SPR spectra, (**d**) sensitivity of the functionalized CNT-based probe for different concentrations of SMX. Reprinted with permission from [91]. Copyright 2018 Springer.

The detection limit improved from 1.137 μM in the case of the enzyme entrapped gel as a sensing layer to 0.8918 μM in the non-enzymatic CNT-based approach. The corresponding response in the two cases, with sensitivity variation along with the desired concentration range, is shown in Figure 20. The maximum sensitivity at the lowest concentration also increased from 0.29 nm/μM for the enzyme-based platform to 0.37 nm/μM for CNTs as sensing material.

**Figure 21:**
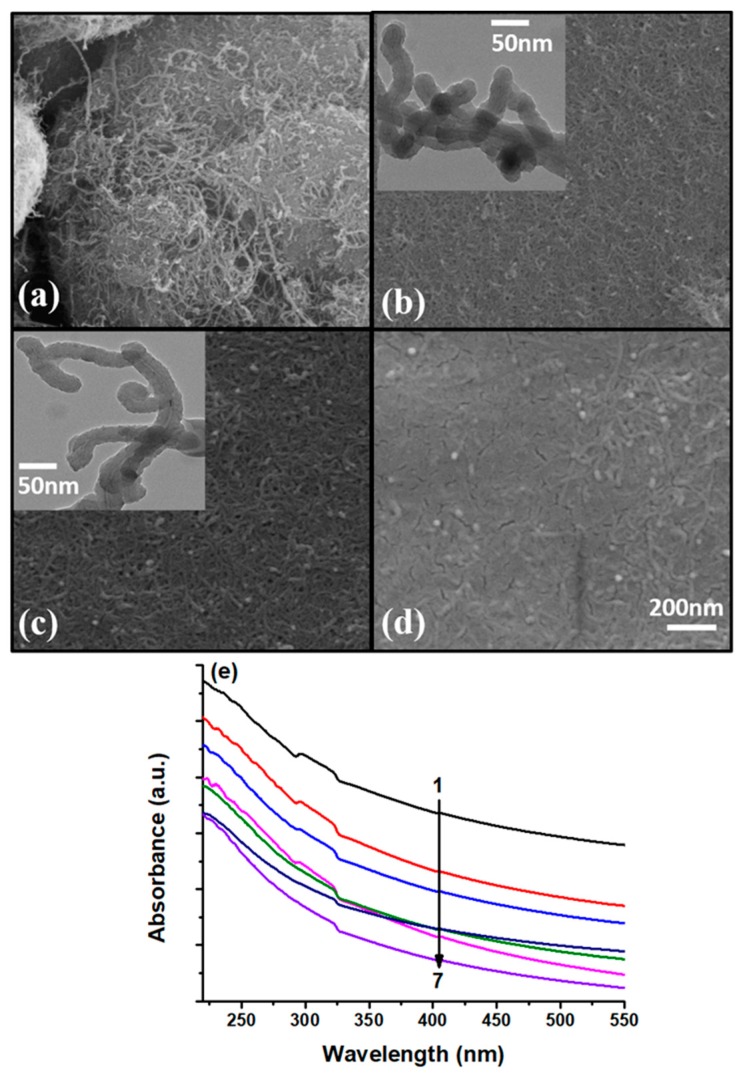
SEM images of (**a**) as obtained MWCNTs, (**b**) DA embedded MWCNTs-PPy matrix, (**c**) DA imprinted MWCNTs-PPy matrix, (**d**) Nafion/MWCNTs-PPy MIP nanocomposite, and (**e**) UV-vis spectra of the MWCNTs-PPy MIP nanocomposite during seven elution steps. Reprinted with permission from [92]. Copyright 2019 Elsevier.

In another biosensor development, CNTs are employed as a matrix for the molecular imprinting (MIP) of dopamine (DA), providing a generic platform for the sensing of neurotransmitters [92]. The MIP on the surface of CNTs encompasses the advantages of high sensitivity and selectivity due to the easy removal and uptake of the analyte during sensing, large surface to volume ratio providing increased sensing hotspots, and a stable composite with imprinting conducting polymer (polypyrrole; PPy) due to binding in a de-localized π-electron cloud of CNTs and PPy. Polypyrrole/CNTs composites were used in the development of several SPR-based sensors due to their unique conductivity, making them favorable for sensitivity enhancement of SPR sensors [86,87]. A permselective nafion membrane was also employed over an MIP layer to minimize the cross-signaling from interfering anions, such as ascorbic acid and uric acid. The morphological characterization of the nanocomposite was confirmed through SEM and TEM images, as shown in Figure 21a–d. The removal of DA for the formation of imprinted sites in MIP layer over MWCNTs was confirmed by UV-Vis spectroscopy, as shown in Figure 21e.

**Figure 22:**
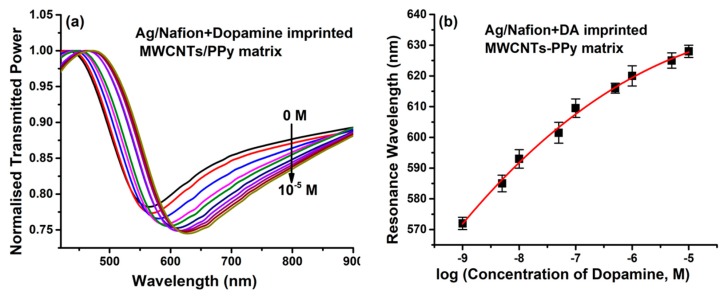
(**a**) SPR plots for various DA concentrations, and (**b**) calibration curve of the sensor. Reprinted with permission from [92]. Copyright 2019 Elsevier.

The SPR response was recorded after optimizing various probe parameters for 0 to 10^−5^ M DA concentration in artificial cerebrospinal fluid, as shown in Figure 22a. A red shift of 69 nm was observed for the whole concentration range, with a non-linear response of the resonance wavelength with DA concentration (Figure 22b).

A similar MIP and SPR-based platform was also reported for the sensing of complex structures, like proteins (BSA). The surface imprinting on CNTs provides an improved platform as compared to bulk imprinting methodologies, where the removal of such complex molecules is cumbersome [93]. Moreover, the catalytic properties of MWCNTs/Cu nanoparticles have been employed in a fiber optic SPR sensor for the effective and fast detection of nitrates in real samples, like soil and river water analysis [94]. The sensing methodology was based on the reduction of nitrate ions to ammonium ions by the catalytic properties of copper nanoparticles and the adsorption of ammonium ions on the CNT surface, bringing a change in SPR response for 10^−6^ M to 5 × 10^−3^ M nitrate concentration.

## 4. Summary

Carbon-based nanomaterials have attracted the attention of the scientific community for the fabrication of plasmonic-based sensors. This is because of their unique electrical, mechanical, chemical, thermal, and optical properties. The role of carbon-based nanomaterials in SPR-based sensors, their current developments, and challenges have been reviewed in this article. In plasmonic-based sensors, carbon-based nanomaterials play various types of roles, such as plasmonic layers, sensitivity enhancement material, and sensing matrix material. In this review, the roles of graphene and its derivatives and carbon nanotubes in sensors have been discussed.
